# Pelvic and muscular metastasis of a renal cell carcinoma: A case report

**DOI:** 10.3892/ol.2013.1172

**Published:** 2013-02-01

**Authors:** CAROLINA D’ELIA, TOMMASO CAI, LORENZO LUCIANI, MARIELLA BONZANINI, GIANNI MALOSSINI

**Affiliations:** 1 Departments of Urology, Santa Chiara Hospital, Trento I-938100, Italy; 2 Pathology and Diagnostic, Santa Chiara Hospital, Trento I-938100, Italy

**Keywords:** renal cell carcinoma, metastasis, muscular, pelvic

## Abstract

We report a case of an uncommon site of metastasis of a renal cell carcinoma. The most common sites of renal cell carcinoma metastasis are the lung, lymph nodes, liver, bone and adrenal glands; skeletal muscle metastasis is a rare occurrence. We report the case of a 75-year-old female who underwent a laparoscopic left radical nephrectomy for a renal neoplasm in 2011. The histological examination revealed the presence of a renal cell carcinoma, Fuhrman grade 2, with extensive necrosis and phlogosis areas (TNM 2009 RCC pT2a). Ten months later, the patient noted an indolent swelling on the proximal third of the right thigh and underwent a ultrasonographic and CT evaluation, documenting the presence of a pathological, solid bulk in the front of the right iliac vessels and in the rectus femoris muscle. The fine needle biopsy revealed a metastasis of renal cell carcinoma. The patient underwent 4 cycles of sunitinib therapy, followed by 3 cycles of salvage therapy with sorafenib, which were well tolerated. This unpredictable behaviour of RCC suggests the need to perform a thorough follow-up of patients.

## Introduction

We report a case of a very unusual site of metastasis of a renal cell carcinoma. Renal cell carcinoma represents 2–3% of all malignancies (1), with an annual increase in incidence of ∼2%. Renal cell carcinoma represents ∼90% of kidney neoplasms (2); 20–25% of the patients initially present with advanced disease and ∼5% present with a single metastatic site (3).

Patients affected by renal cell carcinoma will develop metastasis during the follow-up period in ∼30% of cases (4). The most common sites of renal cell carcinoma metastases are the lung (50%), lymph nodes (35%), liver (30%), bone (30%) and adrenal glands (5%) (5); skeletal muscle metastasis is a rare occurrence.

## Case report

A 76-year-old Caucasian female who was a non-smoker, with a history of heart ischemic disease, hypertension, anemia and diabetes, underwent a laparoscopic left radical nephrectomy for a renal neoplasm of 10×7 cm in the superior left renal pole in 2011. The postoperative course was uneventful and the patient was discharged after 6 days. The histological examination revealed the presence of a renal cell carcinoma, Fuhrman grade 2, with extensive necrosis and phlogosis areas (TNM 2009 RCC pT2a). Informed consent was obtained from the patient.

After discharge, the patient was readmitted for a sciatica episode and underwent two abdomen ultrasonography examinations, with no notable pathological findings.

After a few days, the patient noted an indolent swelling in the proximal third of the right thigh. The patient subsequently underwent ultrasonographic evaluation, revealing the presence of a solid, vascularized mass that was ∼40×22 mm in size.

**Figure 1:**
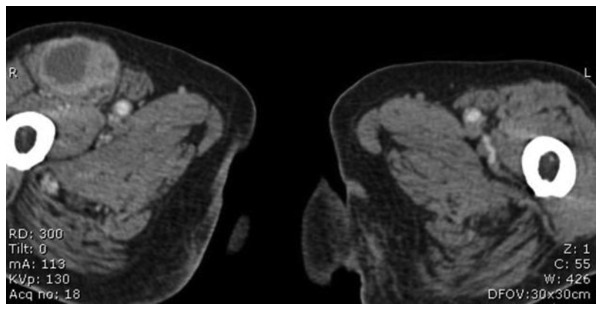
CT scan showing a solid expansive lesion in the rectus femoris muscle.

The subsequent CT documented the presence of a pathological, solid, dishomogeneous bulk that was ∼4.5 cm diameter, in the front of the right iliac vessels, immediately cranial to the inguinal region. Another smaller (1.5 cm) lesion was observed in the ipsilateral inguinale region, while an additional solid expansive lesion was noted in the *rectus femoris* muscle (Fig. 1).

**Figure 2:**
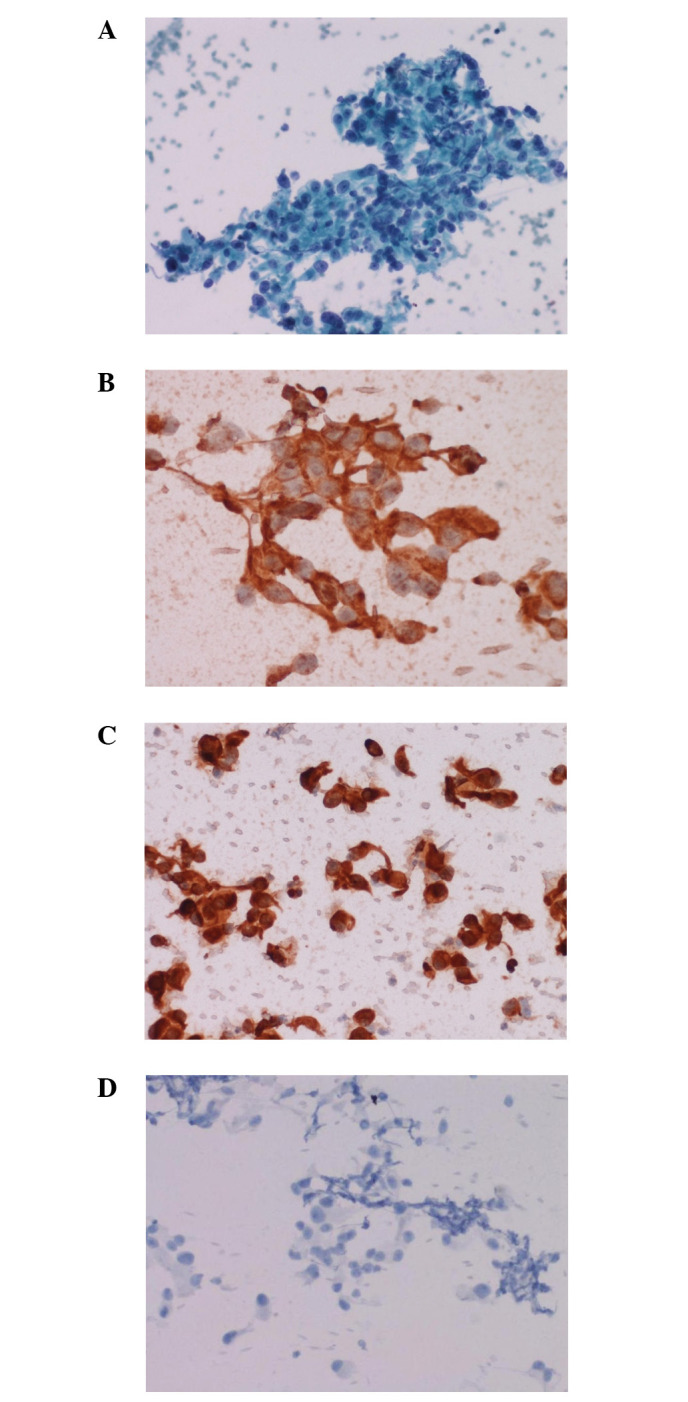
(A) Histochemical assay of fine needle biopsy of the mass (Papanicolaou; magnification, ×200). (B) Histochemical assay of fine needle biopsy of the mass is positive for vimentin and CAM and (D) negative for cytokeratin 7 (magnification, ×400).

The fine needle biopsy of the muscle mass documented a metastasis of renal cell carcinoma (Fig. 2). The histochemical analysis revealed positive staining for vimentin and CAM, and negative staining for cytokeratin 7.

Moreover, we performed a cerebral CT and a total body bone scan, in order to achieve a complete stadiation of the patient. Neither of the examinations revealed further meta-static localization.

A joint evaluation of the patient was performed with the oncologist, the general surgeon and the radiotherapist. With regard to the pathological stage of the disease and the comorbidity of the patient, it was decided to refer the patient for targeted therapy with sunitinib.

Therefore, the patient underwent 2 cycles of sunitinib therapy. Subsequently, due to the onset of edema in the right lower limb, the patient underwent an abdominal CT, revealing a nodal progression of the disease along the right femoral and iliac vessels. The patient underwent 6 further cycles of salvage therapy with sorafenib, which were well tolerated. The subsequent CT scan revealed a lymphonodal progression of disease.

## Discussion

Renal cell carcinoma presents a unpredictable behavior, even after surgical therapy, and the median time to recurrence may be extensive. McNichols *et al* demonstrated that 11% of meta-static RCC cases occurred more than 10 years after the initial diagnosis, even after complete resection (6).

Skeletal muscle metastasis is rare, regardless of the site of origin. A limited number of cases concerning skeletal muscle metastasis have been described in the literature, and its prevalence is ∼1.6% (7). Possibly the first case of skeletal metastasis originating from an RCC was reported in 1979 by Chandler *et al*, describing a slowly enlarging biceps muscle mass as an atypical presentation of RCC, diagnosed with a soft tissue biopsy needle (8). More recently, Ali *et al* reported the occurrence of a persistent left arm swelling accompanied by wrist drop as an atypical presentation of renal cell carcinoma (9).

Several other studies have demonstrated the development of muscular metastasis during the follow-up period after radical nephrectomy, with onset times varying between a number of months and 19 years. Additionally, muscular metastasis has been found in various muscular localizations, such as, respectively, the thigh (at the level of the great adductor muscle or, in our case, the *rectus femoris*), the iliopsoas muscle or the erector spine muscle (10–12).

The mechanism involved in the metastatic spreading to the skeletal muscular tissue is not fully understood. Several explanations have been suggested, such as direct invasion or hematogenous spreading. Moreover, Merimsky *et al* suggested that the relative resistance of the muscular tissue to the meta-static spreading should be investigated further (13).

The surgical resection of a solitary RCC metastasis improves the survival of patients with metastatic RCC. In the present case, with regard to the presence of multiple metastases and the comorbidity, and in accordance with the wishes of the patient, we decided to refrain from administering surgical treatment and the patient was referred to the oncologist.

At present, there is no agreement with regard to a surveillance protocol for patients treated for RCC. Additionally, it is unclear whether an early recurrence diagnosis will improve the survival of the patients (14). However, an early recognition of tumor recurrences means that the therapeutic approach to the disease, whether it be surgical metastasectomy or systemic treatment, through the use of targeted therapies, will be more effective.

The unpredictable behaviour of RCC suggests the need to perform a thorough follow-up of patients, and to investigate all soft tissue masses, including cytopathologically, that develop in patients with a history of RCC.
